# Nanofibrous asymmetric collagen/curcumin membrane containing aspirin-loaded PLGA nanoparticles for guided bone regeneration

**DOI:** 10.1038/s41598-020-75454-2

**Published:** 2020-10-23

**Authors:** Mohammad Ali Ghavimi, Amirhossein Bani Shahabadi, Seyedhosein Jarolmasjed, Mohammad Yousef Memar, Solmaz Maleki Dizaj, Simin Sharifi

**Affiliations:** 1 Department of Oral and Maxillofacial Surgery, Faculty of Dentistry, Tabriz University of Medical Sciences, Tabriz, Iran; 2 Department of Clinical Sciences, Faculty of Veterinary Medicine, University of Tabriz, Tabriz, Iran; 3 Infectious and Tropical Diseases Research Center, Tabriz University of Medical Sciences, Tabriz, Iran; 4 Dental and Periodontal Research Center, Tabriz University of Medical Sciences, Tabriz, Iran; 5 Stem Cell Research Center, Tabriz University of Medical Sciences, Tabriz, Iran

**Keywords:** Drug delivery, Tissue engineering and regenerative medicine, Microbiology, Stem cells, Materials science, Nanoscience and technology

## Abstract

The goal of the current study was to develop an asymmetric guided bone regeneration (GBR) membrane benefiting from curcumin and aspirin. The membrane was prepared using electrospinning technique and then was physic-chemically characterized by the conventional methods. The release profile of aspirin from the prepared membrane was also measured by ultraviolet spectrophotometry. Also, the antibacterial activities of the membrane was evaluated. We also assessed the in vitro effects of the prepared membrane on the biocompatibility and osteogenic differentiation of dental pulp stem cells (DPSCs), and evaluated in vivo bone regeneration using the prepared membrane in the defects created in both sides of the dog’s jaw by histology. The results from the characterization specified that the membrane was successfully prepared with monodispersed nanosized fibers, uniform network shaped morphology, negative surface charge and sustained release platform for aspirin. The membrane also showed antimicrobial effects against all tested bacteria. The presence of curcumin and aspirin in the asymmetric membrane enhanced osteogenic potential at both transcriptional and translational levels. The results of the animal test showed that the test area was completely filled with new bone after just 28 days, while the commercial membrane area remained empty. There was also a soft tissue layer above the new bone area in the test side. We suggested that the prepared membrane in this work could be used as a GBR membrane to keep soft tissue from occupying bone defects in GBR surgeries. Besides, the surgeries can be benefited from antibacterial activities and bone healing effects of this novel GBR membrane while, simultaneously, promoting bone regeneration.

## Introduction

Bone defects are considered among the common devastating problems worldwide. Although bone grafting has been applied in the clinic to solve this issue, it has been reported that the rapid migration of fibroblasts to the defect or fracture site can shape a fibrous capsule which prohibits the efficient growth of blood vessels and osteoblasts into the graft tissue and finally result in bone nonunion^1^. New developed guided bone regeneration (GBR) methods which effectively halts bone destroying and improves new bone generation hold promise in this context^2,3^. Creation and maintenance of a secluded space via a barrier membrane is a fundamental rule in GBR which inhibits invasion of the rapidly growing fibrous capsule and other soft tissues and thus facilitated the inhabitance of host bone-originated osteoblasts in defect niche^4^. High biocompatibility, satisfactory degradation and mechanical specifications in order to produce proper barrier functionalities, space maintenance and clinical handling should be the essential properties of used membranes^4,5^. Enhancing these properties and the ability to complete new bone regeneration via membrane materials is an important research field in bone regeneration.

Nanotechnology is the use of a substance on an atomic, molecular and supra-molecular scale and nanomedicine deals with the medical implementation of nanotechnology. Nanotechnology-based production of asymmetric membranes is considered to be a perfect bone healing which provides a top layer that acts as an inhibitory barrier against pathogen invasion and physical damages and also an inner layer which allows bone resorption and maintains the niche that is crucial for bone regeneration^6,7^.

Electro-spinning as a conventional textile industry using polymer science has lately been introduced as a novel strategy in the production of nanoscale biomimetic scaffolds in tissue engineering. It is also a promising controlled drug delivery system which allows the incorporation of therapeutic agents into the non-woven nanofiber meshes during the electrospinning process^2^.

Collagen as the main constituent of skin, bone, tendon, and connective tissue of animals is an appropriate scaffolding material for tissue engineering areas^8^. Owing to its outstanding biocompatibility and biodegradability, collagen membranes have been applied in the GBR technique and have shown suitable clinical outcomes^9^.

Curcumin, the main bioactive constituent of turmeric, has been recognized and revealed to have a remarkably extensive range of useful biological functions such as anti-microbial, anti-cancer, antioxidant, anti-inflammatory, anti-angiogenic activity^10^. Moreover, it shows beneficial effects in wound healing processes^11–13^. Safety assessment evaluations confirmed that curcumin is very safe as it is tolerated high concentrations without any toxic impacts^14^. According to reports, curcumin has shown promoting bone healing as well. Besides, the local administration of curcumin containing nanomaterials efficiently prevented bone resorption and inflammation related to experimental periodontal disease^15,16^.

Aspirin (acetylsalicylic acid) has been broadly applied for years as a non-steroidal anti-inflammatory drug (NSAID). Recently, some reports have shown that aspirin can accelerate bone repair and may control the balance between bone formation and bone resorption and prevent the differentiation and maturity of osteoclasts^17,18^. It has been reported that aspirin can prevent from invading soft tissue to bone defects in GBR surgeries^19^. Several studies have shown the application of some NSAIDs for repairing bone tissue, particularly in bone healing and the treatment of bone fractures^20^. The study of Cao et al*.* on mini swine calvarial bone defect model displayed that aspirin causes to accelerate the regeneration of bone marrow mesenchymal stem cells (BMSC). Their study exhibited that aspirin at 75 μg/ml stimulated the osteogenesis of BMSC in vivo and in vitro, displayed by new bone volume in the nude mice transplantation model and alizarin red staining (*p* < 0.01), respectively. Treatment of defects with aspirin-BMSC caused better new bone fill significantly at 6 months' post-surgery (*p* < 0.01). The concentration of IFN-γ and TNF-α was reduced significantly with aspirin-BMSC treatment (*p* < 0.05)^21^.

Generally, directly combined drugs into the GBR membranes, which lead to the fast release of the drugs from the membranes, subsequent in a high burst release and short release period^22^. Gastric erosion and hemorrhagic micro-bleeding are side effects of aspirin, which resulted from long-term use of it, but topical and low-dose applications of aspirin for therapy of disease would be very hopeful^23^.

The aim of the current study was to design an asymmetric membrane including aspirin-loaded PLGA nanoparticles and curcumin nanofibers for guided bone regeneration purposes.

## Materials and methods

### Materials

The materials used are specified as in the following: 2,2,2-Trifluoroethanol and Glutaraldehyde (25% Aqueous Solution) purchased from Merck (Darmstadt, Germany). Fetal bovine serum (FBS), Dulbecco’s modified Eagle’s medium (DMEM), Dulbecco’s phosphate buffer saline (DPBS), trypsin, penicillin G and streptomycin were achieved from Gibco (Life Technologies, Carlsbad, CA, USA). poly lactic-co-glycolic acid (PLGA (75:25), Acetylsalicylic acid (Aspirin), curcumin, collagen (Bovine type), Dimethylsulfoxide (DMSO), MTT (3-[4,5-dimethyl-thiazol-2-yl]-2,5-diphenyl-tetrazoliumbromide), paraformaldehyde, glycerol phosphate, ascorbic acid, dexamethasone, RIPA buffer, protease inhibitor cocktail, Alkaline Phosphatase Assay Kit, QuantiPro BCA Assay Kit were bought from Sigma-Aldrich (Darmstadt, Germany). Pluronic F68 was obtained from MPBIO (Illkirch Cedex, France), RNA extraction kit was purchased from GeneAll (Seoul, Korea). DNase I was obtained from Thermo Fisher Scientific (Hamburg, Germany). HOT FIREPol EvaGreen (no ROX) qPCR mix kit and cDNA synthesis kit obtained from Solis Biodyne (Tartu, Estonia). Mouse anti-rabbit osteocalcin (OCN), Runx-2 and GAPDH primary antibodies as well as goat anti-rabbit secondary antibody were purchased from Santa Cruz Biotechnology (Santa Cruz, CA, USA). Western Blotting Luminol Reagent and Blotto solution were purchased from Santa Cruz Biotechnology (Santa Cruz, CA, USA). All the primers were purchased from Metabion (Lower Saxony, German). The dental pulp stem cells (DPSCs) were purchased from Dental Research Center, Tehran, Iran. All solvents were acquired at lab-grade purity and utilized without further purification.

### Methods

In this work, we prepared an asymmetric GBR membrane. Collagen nanofibers (CNFs) including PLGA-aspirin nanoparticles (PANPs) as was one side of the asymmetric membrane (PACNFs), and curcumin/collagen nanofibers on the other side of the asymmetric membrane (CCNFs).

### Production of aspirin-loaded PLGA nanoparticles

In the first step, 100 mg PLGA was dissolved in acetic acid and water. Then, 3 mg of aspirin was added to anhydrous ethanol to produce a saturated solution and then individually added to the PLGA solution under magnetic steering of 100 rpm at 25 °C (solution 1). In a separate beaker, 50 ml pluronic F68 was added into 50 ml dichloromethane under magnetic steering of 100 rpm at 25 °C (solution 2). The solution 1 was then added into solution 2 drop by drop and for 10 min was stirred (450 rpm, 25 °C). The obtained suspension was repeatedly washed with distilled water and then transformed into freeze-dried at (− 55 °C and for 24 h).

### Fabrication of asymmetric membrane

Collagen solution in 2,2,2-Trifluoroethanol (8% w/v) was prepared. Passive loading of PANPs (adding the nanoparticles in the polymeric solution prior to electrospinning) was used for the fabrication of PANPs-loaded collagen nanofibers. For this, 10 mg of the prepared PANPs was suspended in 10 ml of collagen solution under constant stirring in dark conditions. Then, the obtained suspension was electrospun by electrospinning technique (ANSTCo RN/I, Iran). A capillary tube in a vertical form was used. A collector also was used to collect the nanofibers over the electrospinning jets (20 kV voltage, distance of 10 cm between a collector and a capillary tube and 1.5 ml/h flow rate). A white nanofibrous sheet was formed on the collector.

For the preparation of collagen/curcumin side, 5 mg curcumin and 1 g collagen were dissolved in 2,2-Trifluoroethanol. A yellow solution was obtained. In the next step, the yellow solution was electrospun using a similar electrospinning process. The prepared membrane was cut in 1 × 1.5 cm and was stored for more investigations.

### Characterization

The mean size for PANPs and the mean fiber diameter of the nanofibers of the membrane (both sides) was measured by Dynamic Light Scattering (DLS) method (Malvern, United Kingdom) at 25° C. Furthermore, SEM images (SEM, TESCAN, Warrendale, PA) were acquired to assess the morphology of the PANPs as well as electrospun nanofibers in both side of the membrane. For SEM images, the prepared nanomaterials were fixed via an adhesive tape on aluminum stubs and covered with gold before the examination. Transmission electron microscopy (TEM) was used to confirm the presence of PANPs in the matrix of CNFs ([TEM], JEM-2100F; JEOL, Tokyo, Japan). Enough low electron density was used in order not to melt the polymers or destroy nanofibers structure.

Measurements of the zeta potential were done using a zeta-sizer (Malvern, UK) at 25 °C in order to evaluate the surface charge of the nanomaterials. For this, the suspension of nanomaterials was diluted with water and injected into the capillary cell of zeta-sizer. The qualitative comparison of crystalline state of the components of the material was examined by the X-ray diffraction (XRD) technique (Philips TW 1710 diffractometer with Cu-Kα incident radiation regulated at 40 kV and 30 mA). The records were done at room temperature over the 2θ range of 20°–60°, with scanning rate 3˚/min. Besides, to get the molecular structure and inter/intra molecular bonding of the prepared nanomaterials, Fourier transform infrared spectroscopy (FTIR) was used (Thermo Scientific FTIR (Thermo Nicolet-6700) spectrophotometer) in the range 4000–400 cm^−1^.

### In vitro release of PANPs from the asymmetric membrane

To assess the release outline of aspirin from the prepared membrane, a small piece of the asymmetric membrane was put under magnetic steering in 300 ml of phosphate buffer medium (100 rpm, pH of 7.4, and temperature of 37° C). At seven time points (1, 5, 10, 15, 20, 25 and 30 days), 2 ml of the medium was collected and then replaced with the same volume of the primary buffer medium without aspirin. The amount of aspirin was then measured by ultraviolet spectrophotometry at 280 nm (n = 3) for each point. Aspirin concentration was calculated using a standard curve and the release pattern was illustrated.

### Microbial test

Antibacterial effects of the prepared membrane were evaluated by disk diffusion agar on the *Muller-Hinton agar against Staphylococcus aureus (S. aureus), Escherichia coli (E. coli) and Enterococcus faecalis* (*E. faecalis*). Briefly, the bacterial cell suspension equivalent to the 0.5 McFarland standard (1.5 × 108 CFU/mL) was inoculated on the Muller-Hinton plates using a swab and allowed to dry for 10 min. Six millimeters disk-shaped membranes were placed on the agar surface and plates were incubated at 35 °C for 24 h aerobically. Collagen nanofibrous membrane (without any potential ingredient) was used as control disk. The inhibition zones around the disk were measured and considered as representing of the antibacterial activity of tested membranes.

### Evaluation of biocompatibility; in vitro

The sterilized membranes were cut into discs with a diameter of 0.32 cm^2^ to match the 96-well plate size and pre-wetted with the complete culture medium (i.e., DMEM with 10% FBS, 100 mg/mL of streptomycin and 100 U/mL of penicillin), and then the cells were added. Next, we seeded 5000 DPSCs onto the membranes in 200 μL of the medium and incubated the membranes at 37 °C in a 5% CO_2_ atmosphere. The wells’ original culture medium was replaced at different times after culture (24 h, 72 h, and day 7), with MTT (50 μL/well, i.e., 2 mg/mL) in addition to fresh medium (150 μL/well). Then, the plates were incubated for 4 h at 37 °C in the 5% CO_2_ atmosphere. Afterward, we replaced the 200 μL/well of DMSO with the wells supernatant for 30 min. Next, 100 μL of the solution was moved to another 96-well plate and the absorbance was determined at 570 nm using a Microplate Reader.

### Cell differentiation

We seeded DPSCs (5000 cells/cm^2^) in 6-well plates on each sterile membrane and allowed them to time for cell adherence on the membranes surface through incubating at 37 °C and 5% CO_2_ in basal medium for 24 h. Then, the basal medium was exchanged with osteogenic medium (i.e., the basal medium which is supplemented with 0.2 mM ascorbic acid, 10^−8^ M dexamethasone, and 10 mM β-glycerolphosphate). We refreshed the osteogenic medium every 72 h for 21 days—we cultured DPSCs in the osteogenic medium as control groups.

### RNA extraction and cDNA synthesis

On day 21, we extracted the total RNA using an RNA extraction kit following the kit’s protocol (GeneAll, Seoul, Korea). To eliminate the genomic DNA, we used DNase I was according to the manufacturer’s protocol. Next, the extracted RNAs were normalized based on their RNA content, which was determined by a NanoDrop spectrophotometer (ND-1000, Thermo Fisher Scientific, MA, USA). We used cDNA synthesis kid according to the kit manual for cDNA synthesis, using a LightCycler instrument (Roche Applied Science, USA).

### Gene expression

We used a real-time polymerase chain reaction (qRT-PCT) kit to evaluate gene expression. We used the following primers: (housekeeping; 5′-AGCCACATCGCTCAGACAC-3′, 5′-GCCCAATACGACCAAATCC-3′), OCN (late marker of osteogenesis; 5′-AGCAAAGGTGCAGCCTTTGT-3′, 5′-GCGCCTGGGTCTCTTCACT-3′), and RUNX2 (early marker of osteogenic differentiation; 5′-ATCTGAGGTAACTTGCTAACG-3′, 5′-CCGAAGTCAACATATCAATACAC-3). The 2^−∆∆CT^ method was applied to calculate the Fold-change expression of the target genes.

### Biochemical assays

At three points on day 7, day 14, and day 21, we drained the osteogenic medium, and to eliminate the serum proteins, we submerged the DPSCs seeded membranes for 8 h in serum-free DMEM, after that, we conducted DPBS washing three times. The lysis buffer (i.e.,10 mM Tris which was supplemented with 0.2% triton in PBS) was used for DPSCs lysis. This lysate was used to evaluate the activity of ALP employing ALP assay kits.

### Western blotting

The RUNX2 and OCN protein expression was quantified, as osteogenic markers using Western blotting at day 21. In short, after serum protein elimination, we lysed the DPSCs-seeded membranes using a RIPA buffer which was supplemented with a protease inhibitor cocktail. We run the lysates with the same level of total protein (as measured using a BCA kit) through 7.5% SDS-PAGE gel. Next, the separated proteins were transferred to a nitrocellulose membrane. We blocked the membranes using 2% non-fat dry milk in 0.1% TBS-T (Tris-buffered saline/Tween-20). Afterward, we incubated the nitrocellulose membranes in primary, and horseradish peroxidase (HRP) conjugated secondary antibodies. Then, we used Clarity Western ECL Substrate (Bio-Rad, USA) to visualize the bands of the membrane. A commercial molecular weight marker (Thermo Scientific, USA) was used to identify the protein bands. The Image J software package was used to determine the density of each band. Also, the relative density of the target proteins was normalized to GAPDH.

### In vivo bone regeneration behavior

To study the bone regeneration performance of the prepared membrane, a model of alveolar bone defect using 6 adult mongrel dogs were performed. Dogs were anesthetized by intramuscular injection of 10 mg/kg ketamine 10%, 0.1 mg/kg acepromazine 2% and Coktail IM. A defect was generated (8 mm in diameter and 4 mm deep) on both sides of the dogs’ jaw with a 6 mm trephine drill, which was cooled while washing with sterile saline. One side as test side for insertion of the prepared membrane (15 mm in diameter) and the other side as control side. In the control side, the defects were covered with the commercial membrane (Regen collagen, 15 mm in diameter). The periosteum and skin were sutured using 5-0 monofilament vicryl suture in an interrupted fashion.

On day 28, we anesthetized the dogs for surgery. The samples were immobilized in a hydrochloric acid solution of formaldehyde. Then, we decalcified them in 17% EDTA solution and dehydrated them using an ascending graded alcohol series. Lastly, we fixed them in paraffin. A series of cross-sections with a diameter of 5 μm in the center of bone defects were prepared. Then, we made them by hematoxylin and eosin (H&E) staining for investigation under light microscopy (BX51, Olympus, Japan). The institutional ethics committee of the Tabriz University of Medical Sciences agreed with the use of animals for the experiments conducted in this study, and all experimentations were done based on the related guidelines and rules.

### Statistical analysis

All data were stated as the mean ± standard deviation. To show statistical significance, *p* < 0.05 was considered.

### Ethical considerations

The institutional ethics committee of the Tabriz University of Medical Sciences agreed with both theses and all experimentations were done based on the related guidelines and rules. The code of ethics for preparation and physicochemical assessments was IR.TBZMED.REC.1397.317. For cellular and in vivo investigations the code of ethics was IR.TBZMED.VCR.REC.1399.164.

## Results and discussions

### Electrospinning set up

**Figure 1:**
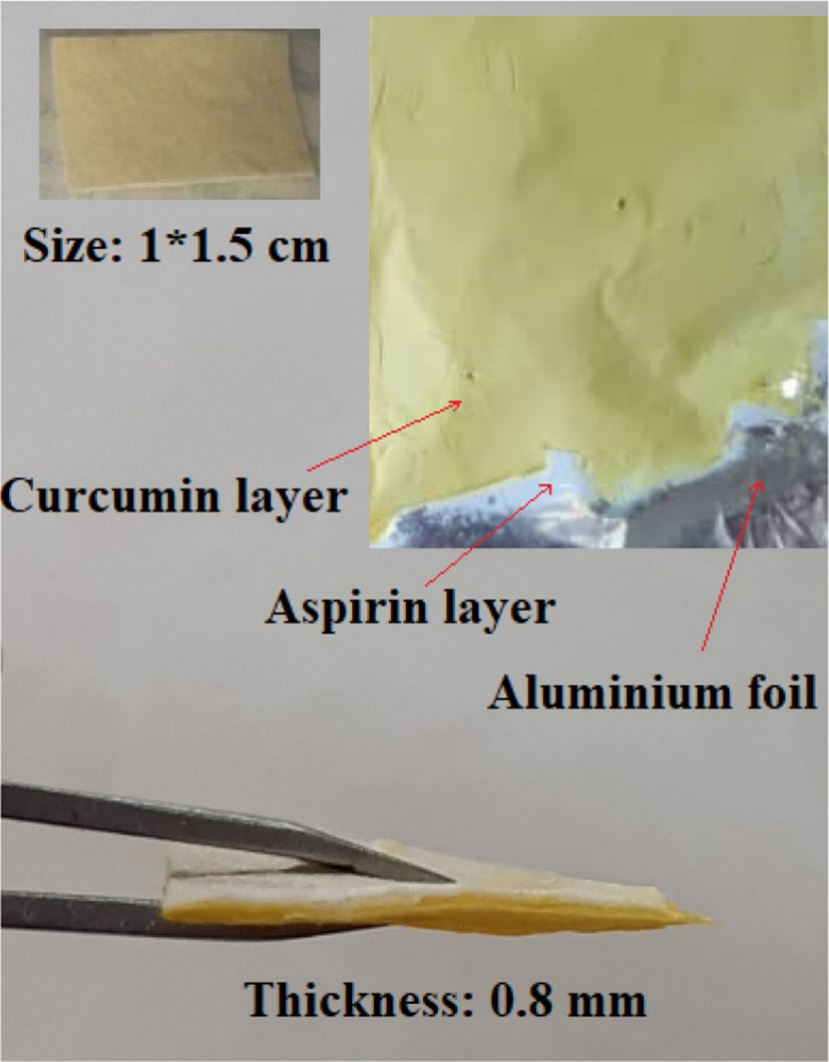
The pictures for the prepared membrane.

The shape and the size of the nanomaterials should be well-ordered, which is not a simple procedure. The growing of particles from atoms to nanometric or micrometric sizes can be managed to yield mono-dispersed sizes^6,24^. Electro-spinning as a quickly developing technique displays the ability to form varied morphologies due to its functionality, flexibility, and simplicity. Processing variables for the electrospinning process including needle to collector distance, voltage, flow rate, also, solution parameters such as surface tension, viscosity, and electrical conductivity of the solution can control the morphology of the nanofibers^8^. The goal of electrospinning procedure optimization is to identify the conditions of producing fibers with a minimum diameter^25^. Optimum settings in our study were: 20 kV voltage, distance of 10 cm between a collector, a capillary tube 1.5 ml/h flow rate. In this study, we used the passive loading of PANPs into collagen nanofibers (adding the nanoparticles in the polymeric solution prior to electrospinning). The results of other investigators showed that polymeric nanoparticles, nanotubes, micelles, lipid nanoparticles and etc. can be embedded with electrospun nanofibers to improve different parameters like release outline, drug safety, drug-loading efficiency, and better functionality of the fibers^26–29^. Nanofibers embedded with nanoparticles are similar to co-axial nanofibers as they keep drug safe from organic solvents in the production period and extend the period of drug delivery^30^. Furthermore, it is a more simple technique than co-axial electrospinning because it applies a single nozzle. Figure 1 shows the pictures for the prepared membrane.

### Physicochemical properties

**Figure 2:**
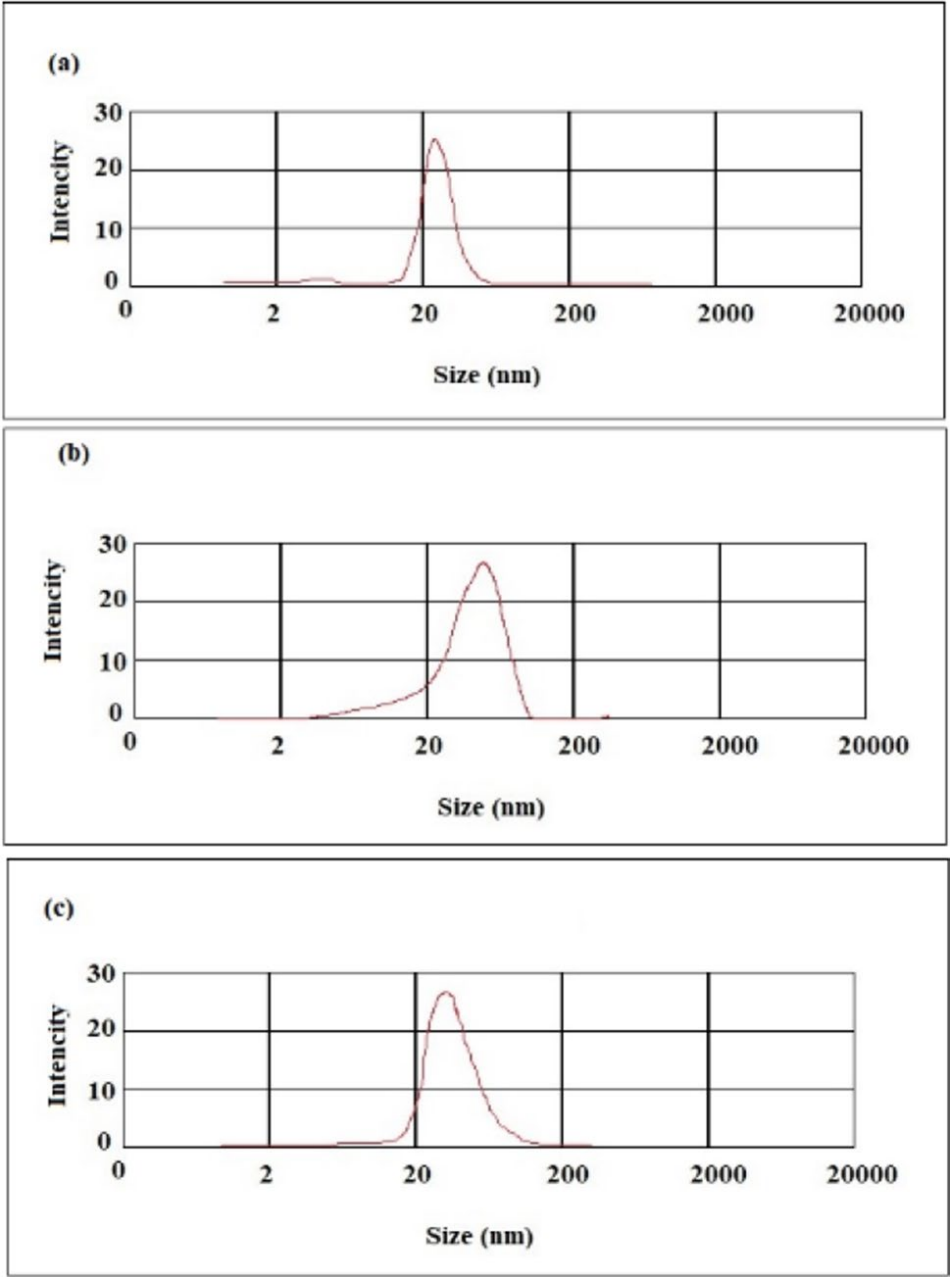
Size distribution of the prepared membrane: (**a**) aspirin-loaded PLGA nanoparticles, (**b**) nanofibrous asymmetric membrane (CCNFs side), (**c**) nanofibrous asymmetric membrane (PACNFs side).

To assure from the prepared nanocarriers suitability for different uses, they should be characterized. The important physicochemical properties of nanomaterials are the mean particle size and point diffraction interferometer (PDI), which affect the endocytosis-dependent cellular uptake^31^. The mean diameter of nanofibers is a main and key parameter that can influence on different properties of nanomaterials in biomedical uses. With respect to the particle size distribution, PDI values more than 0.7 shows that the nanomaterial has a very wide distribution of particle size and is maybe unsuitable to be analyzed by DLS method^32^. The size distribution of the prepared nanomaterials is presented in Fig. 2. The results obtained from DLS showed a mean size of 50.44 nm (PDI of 0.28) for PANPs, 98.52 nm (PDI of 0.49) for PACNFs and 84.06 nm (PDI of 0.39) for CCNFs. The obtained results display the production of relatively mono-dispersed nanomaterials.

**Figure 3:**
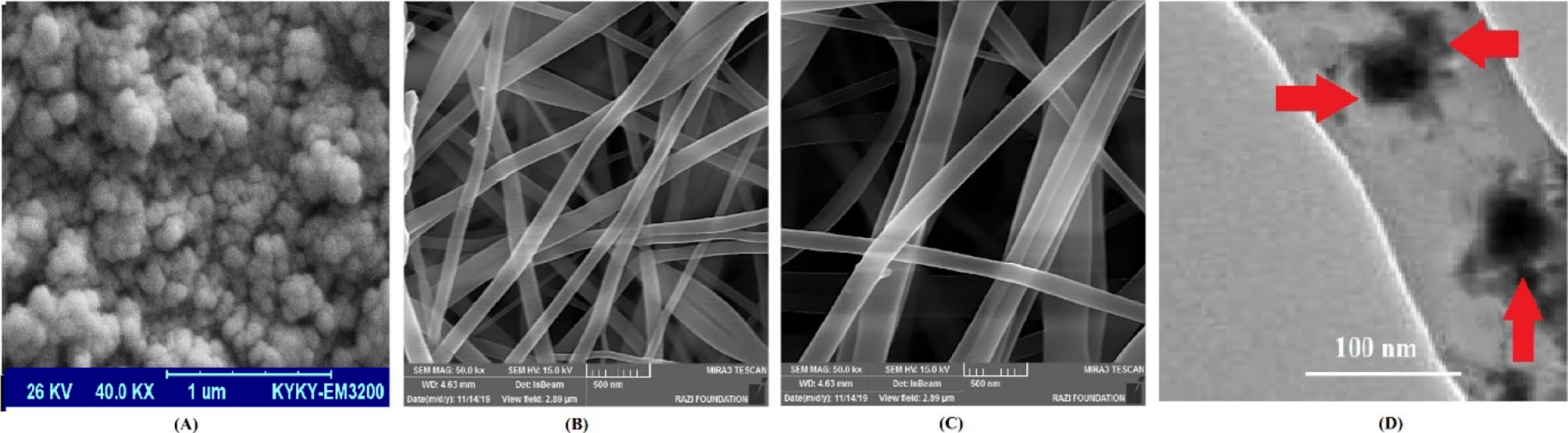
Microscopic images of the prepared membrane: (**A**); aspirin-loaded PLGA nanoparticles, (**B**); the asymmetric membrane (CCNFs side), (**C**); the asymmetric membrane (PACNFs side), (**D**); TEM image of the presence of PANPs in the matrix of the CNFs side.

SEM results displayed uniform agglomerated spherical morphology for PANPs (Fig. 3A). For the prepared nanofibrous asymmetric membrane, SEM results showed successfully formation of nanofibers (Fig. 3B, C). It can be noticed from the SEM results that the nanofibers were randomly electrospun on the collector with high similarity to the construction of the ECM. Furthermore, the nanofibers displayed a network-shaped construction without the presence of beads. Biomimetic nanofibrous scaffolds simulate important properties of the natural ECM, so offer a desirable approach to restore functions or attain desirable responses for tissue regeneration. By modifying the electrospinning parameters controllable fibrous structures can be successfully produced and so offer excellent prospects for the production of biomimetic materials^33,34^.

The anisotropic architecture of natural ECMs is critical for tissue function^35^. Hence, a well-defined architecture is essential for accurately imitate natural ECM for tissue regeneration or guiding cell growth^36,37^. For this purpose, electrospun nanofiber scaffolds with numerous alignments such as radially aligned, yarn, axially aligned have displayed excellent capability in guiding cell migration, shaping cell morphology, and affecting cell differentiation compared with the other kinds of scaffolds both in vivo and in vitro^38–40^. More significantly, specific cellular activities such as cell differentiation, adhesion and migration may lead to promising adaptation of cells in this fibrous membrane as nanoscale microenvironment^41^.

SEM images sometimes does not show the existence of nanoparticles in the matrix of another polymer. Then, it should be checked using TEM. TEM image (Fig. 3D) showed the presence of PANPs in the matrix of nanofibrous asymmetric membrane. As it can be observed, PANPs deposited as spherical entities along the nanofibers. In TEM, the image formation is related to the scattering of electrons by materials of differing density. The higher the density, the darker the TEM image and the lower the density, the brighter the image^42^. Then, due to the higher density of PLGA (75:25) than collagen, the PANPs are darker in TEM image.

**Figure 4:**
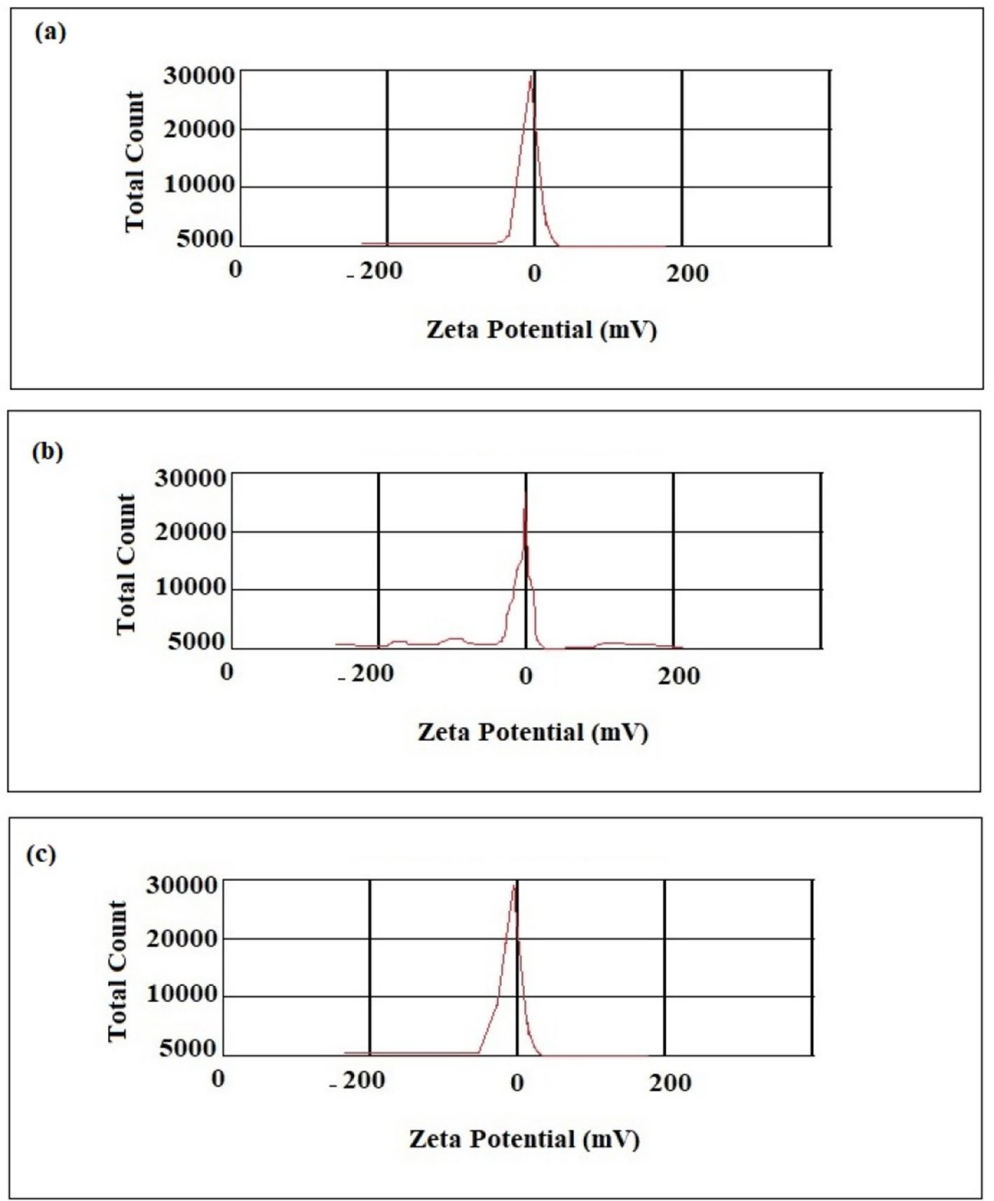
Zeta potential of the prepared materials; (**a**) aspirin-loaded PLGA nanoparticles (PANPs), (**b**) nanofibrous asymmetric membrane (CCNFs side), (**c**) nanofibrous asymmetric membrane (PACNFs side).

Zeta potential measurements (Fig. 4) showed a negative surface charge for all nanomaterials (− 25.92 mV for PANPs (Fig. 4a), − 32.20 mV for CCNFs (Fig. 4b) and − 37.74 mV for CNFs (Fig. 4c)). The surface charge of the particles has main role on their properties. Zeta potential is one of the key parameters usually utilized to estimate suspension stability. As a general rule in stability reports, a value of zeta potential above ± 60 mV shows outstanding stability, while zeta potential less than ± 5 mV generally consequences in particle aggregation. The values between this range results good stability or satisfactory short-term stability. It shows the repulsive charge between the fibers is enough to evade agglomeration^43,44^. Then, the prepared nanomaterials indicated good stability. It has also been informed that the negative zeta potential shows a significant promising impact on the attachment and proliferation of the bone cells. The reports showed that a surface with a negative charge is more available for the attachment and proliferation of osteoblasts than neutral or positive charges^45–47^.

**Figure 5:**
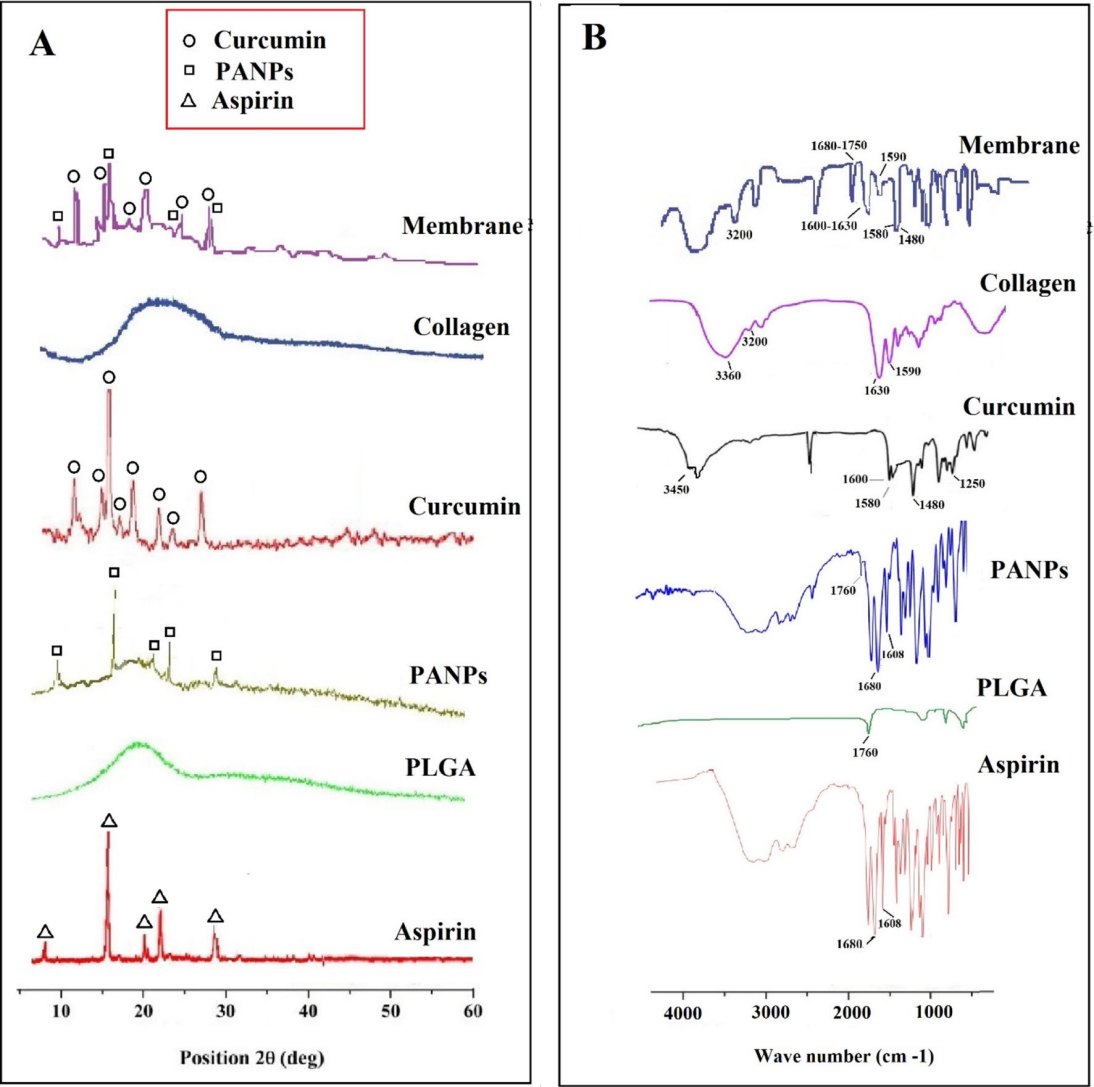
X-ray diffraction pattern (**A**) and FTIR patterns (**B**) for; aspirin, PLGA, aspirin-loaded PLGA nanoparticles, curcumin, collagen and asymmetric membrane.

The X-ray diffraction (XRD) method of the fabricated nanomaterials were measured to confirm the production of the materials and for the qualitative comparison of crystalline state of the costituents of the material. The results for the XRD pattern displays the existence of sharp and high intensity peaks for the prepared aspirin-loaded PLGA nanoparticles as well as the asymmetric nanofibrous membrane (Fig. 5A). The appearance of diffraction two-theta (2θ) peaks at 7°, 15°, 20°, 22° and 27°, corresponding to the characteristic crystal form of aspirin as reported in the literature^48^. Aspirin-loaded PLGA nanoparticles showed all characteristic peaks of aspirin but appear less intense as compared to pure aspirin^48^ which is due to the amorphous nature of the PLGA^49,50^. The XRD pattern of curcumin displayed sharp and intense peaks between 10° and 30°, representing a crystalline arrangement. The major XRD diffraction peaks of curcumin were detected at angles of 9°, 14°, 17°, 18°,21°, 23°, 24° and 25°^51,52^. The XRD diffractogram of the membrane displayed a typical amorphous peak at 20°, which implied that neither collagen nor PLGA could not be crystallized in the course of electrospinning and gave an amorphous structure in the nanofibers. The membrane also showed a reduction in the number of peak intensity for curcumin and aspirin-loaded PLGA nanoparticles (as compared to their pure peak), indicating decreased crystallinity or changes into the amorphous phase of the material during the electrospinning process^9,53^. The obtained outcomes are similar to the earlier study on the collagen–chitosan fibers that according to the authors it could be due to the cooperative effect of the solvent and electrospinning procedure on the chitosan and the collagen^54^.

To evaluate the molecular structure of the prepared nanomaterials, FT-IR was applied. FT-IR peaks (Fig. 5B) showed all characteristic peaks of the prepared nanomaterials with no additional peaks. Four characteristic absorption peaks at 1680 (carbonyl group), 1608 (benzene ring) and a broad peak in the range 2500–3500 cm^−1^ (carboxylic acid group) were observed in the aspirin sample and were also present in the aspirin-loaded PLGA nanoparticles as well as membrane confirming the presence of aspirin-loaded PLGA nanoparticles within asymmetric membrane^55,56^. The spectrum of PLGA showed the C=O absorption band at 1760 cm^−1^ which was also seen in aspirin-loaded PLGA nanoparticles as well as asymmetric membrane^52,57^. Curcumin showed the characteristic intensities of O–H stretching at 3450 cm^−1^, the aromatic C=C stretching at 1580 and 1480 cm^−1^, C=O stretching at 1600 cm^−1^, aromatic C–O stretching at 1250 cm^−1^, aliphatic C–O stretching at 1180 cm^−1^ and =C–H bending at 980 and 900 cm^−1^. C=O stretching appeared at low frequency due to enolization and intermolecular H-bonding^52^. In the FTIR pick of collagen, the main functional groups of collagen were detected. The main peaks were amide I (1680–1620 cm^−1^), amide II (1580–1480 cm^−1^), and amide III (1300–1200 cm^−1^). The strong hydroxyl band in the region of 3200–3600 cm^−1^ was observed to be overlapped with the amide A band (3360–3320 cm^−1^)^58^.

### The release of aspirin in vitro

**Figure 6:**
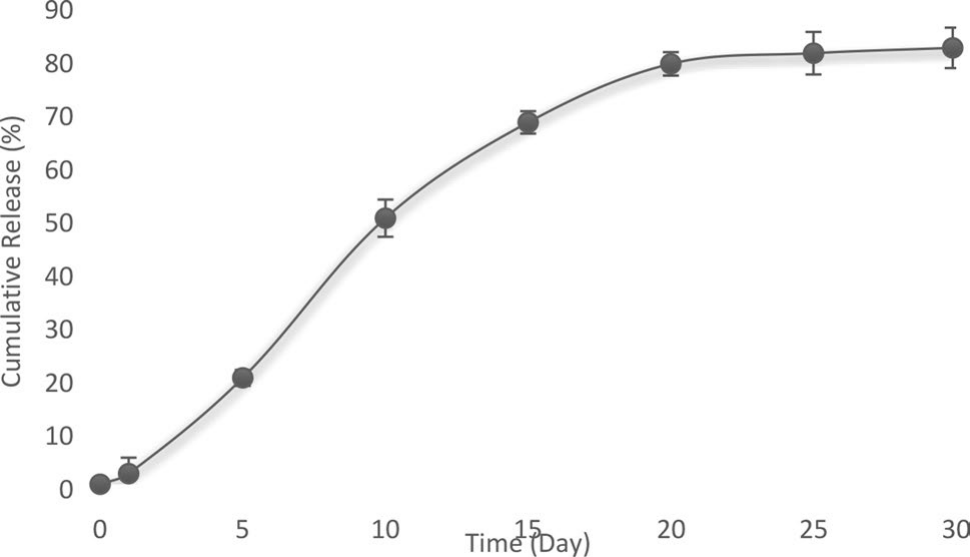
Release curve of aspirin from the asymmetric membrane at 1, 5, 10, 15, 25 and 30 days.

An aspirin-loaded nanoparticle has been produced and applied to determine that aspirin can be released in a sustained form in vitro (Fig. 6). The calibration curves of the absorbance of aspirin at 280 nm displayed an R^2^ = 0.996. A burst release of aspirin was obtained for first to fifth days followed by a sustained release until day 30.

Diffusion and polymer degradation are two main mechanisms of drug release from the polymer matrix^59^. According to Fig. 6, aspirin showed a controlled release profile from the asymmetric membrane. Then, the aspirin not only diffuses through PLGA nanoparticles, but also through the collagen membrane. The initial release concentration of aspirin from the asymmetric membranes was higher (20% for first to fifth days), which was due to the drug diffusion from the surface of the nanoparticles and subsequently from the surface of the asymmetric membrane. Furthermore, late in the release profile, the aspirin concentration was lower but aspirin has sustained-release until the 30th day. It can be suggested that the release may be because of both the diffusion of aspirin from the surfaces of the nanoparticles and the asymmetric membrane and the degradation the matrix of the asymmetric membrane^59^.

### Microbial test

**Figure 7:**
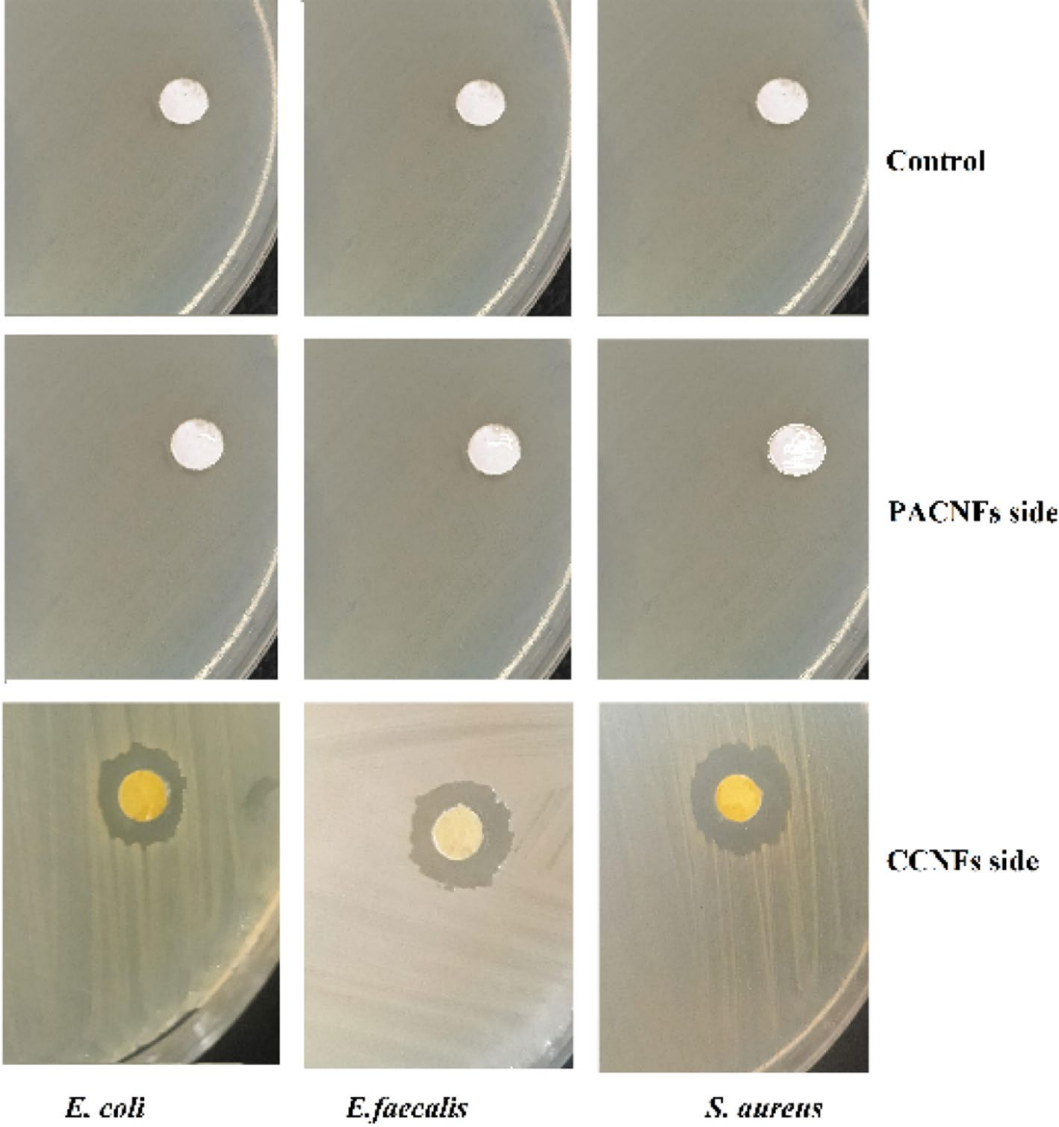
The antibacterial effects of the membrane against *S. aureus*, *E. faecalis* and *E. coli* by detecting the growth inhibition of bacterial isolates around the membrane disks.

The antibacterial effects of the membrane was evaluated against *S. aureus*, *E. faecalis* and *E. coli* by detecting the growth inhibition of bacterial isolates around the membrane disks. The membrane was more effective against *S. aureus* followed by *E. faecalis* and *E. coli* (Fig. 7). The antimicrobial effects of nanocurcumin have been reported against both Gram-negative and Gram-positive bacteria^60^. However, it is reported that the nanocurcumin is more effective against Gram-positive bacteria than Gram-negatives bacteria^61,62^. There was not an observable inhibition zone around the disk of PACNFs side as well as control disk.

### Cellular studies

#### The DPSCs proliferation

**Figure 8:**
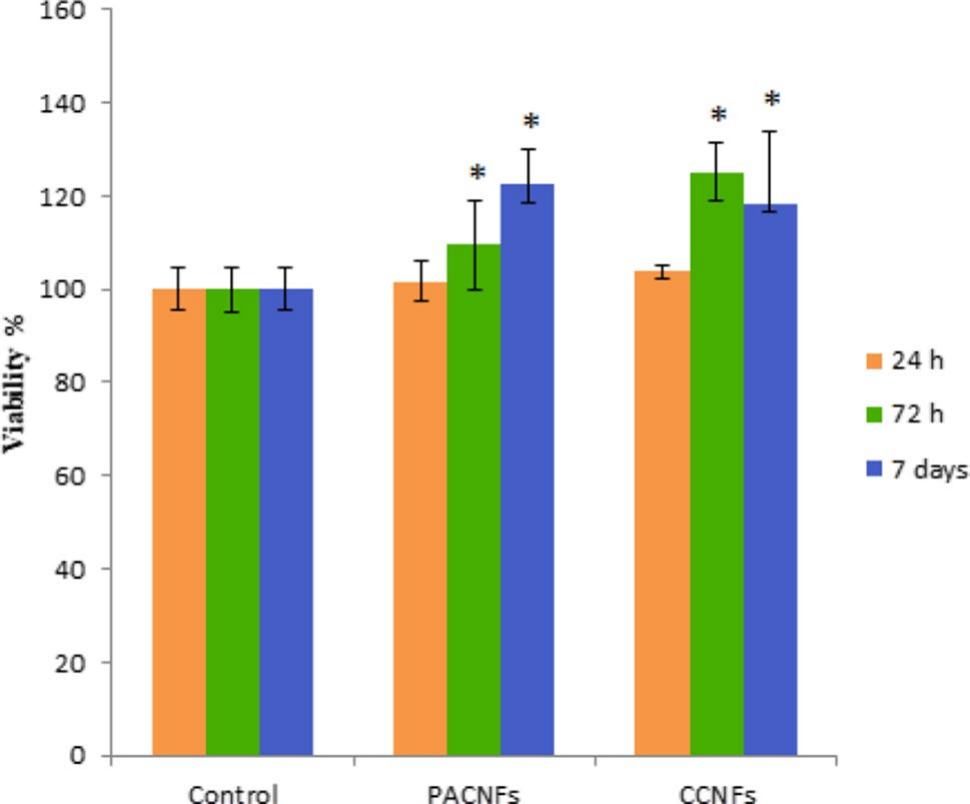
Proliferation of DPSCs cultured on PACFNs and CCNFs membranes at 1, 3, 7 days. An asterisk represents a statistically significant difference (*p* < 0.05) between the test and control group at each time point.

The results of the DPSCs proliferation or metabolic activity (Fig. 8) shown that the cells were metabolically active on PACFNs and CCNFs membranes. Throughout the 72 h and 7 days of culturing DPSCs. The metabolic activity (proliferation) of DPSCs on PACNFs membrane was found to be higher than CCNFs membrane. This increase in proliferation of DPSCs can be partially related to the proliferative effect of aspirin and curcumin in PACNFs and CCNFs membranes, respectively^19,63,64^ (Fig. 8).

#### Osteogenic differentiation

**Figure 9:**
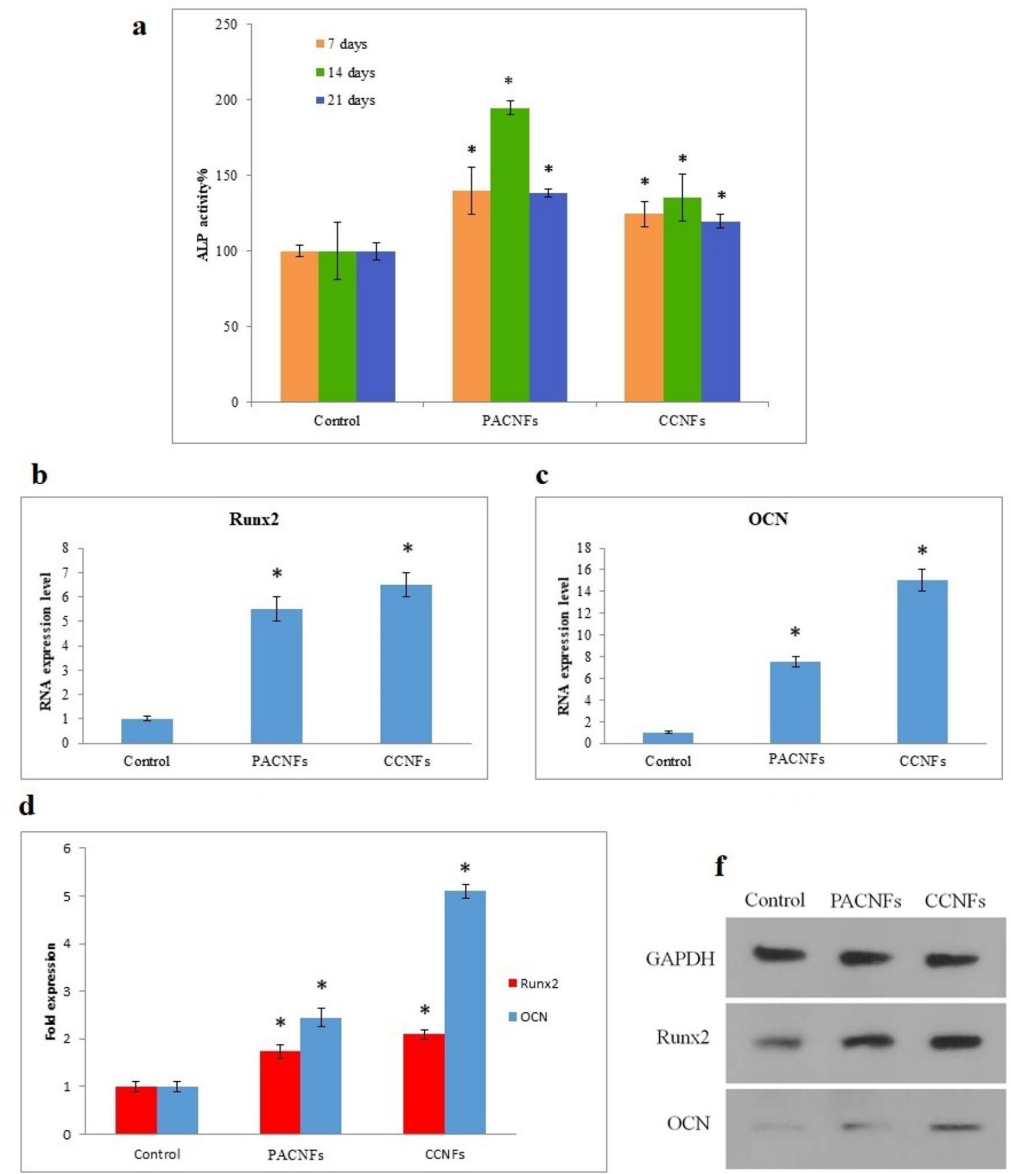
Osteogenic differentiation of DPSCs cultured on CCNFs and PACNs membranes at days 7, 14, and 21 in the osteogenic medium: activity of ALP (**a**); sexpression of genes [Runx-2 (**b**), and OCN (**c**)]; expression of proteins OCN and Runx-2 at day 21 compared to the expression of GAPDH (**d**); bands of western blotting for OCN (6 kDa), Runx-2 (55 kDa), and GAPDH (37 kDa) (**f**). We used three different gels to visualize the expression of OCN, Runx-2, and GAPDH proteins relative to each other (while keeping similar exposure parameters and acquisition settings). An asterisk shows a significant difference (*p* < 0.05) between the control and test groups.

ALP activities of DPSCs seeded on the membranes followed its common trend in osteogenesis^65^ (an increase from day 7 to 14 related to osteogenic differentiation followed by a reduction from day 14 to 21 related to the initiation of the mineralization procedure (Fig. 9a). The normalized amount of ALP activity, which has a main role in mineralization, increased in both PACNFs and CCNFs membranes. An increase in the ALP activity of mesenchymal stem cells in the presence of aspirin and curcumin has also been reported in the previous studies^66,67^.

PACNFs membrane can upregulate Runx-2 and OCN, as osteogenic genes, according to their expression (Fig. 9b, c). Results demonstrated that the aspirin and curcumin significantly affected the DPSCs differentiation (*p* < 0.05). Based on the statistical analysis, there is a significant increase (*p* < 0.05) for the expression of *OCN* and *Runx-2* genes at day 21. The western blot analysis of Runx-2 and OCN proteins on day 21 (Fig. 9d, f) confirm the PACNs significantly higher osteogenic potential in the stimulation of osteogenic differentiation at the level of translation in DPSCs (see also Supplementary Information). Thus, it may be stated that aspirin and curcumin can both translationally and transcriptionally improve the osteogenic differentiation potential in PACNs and CCNFs fibers. Aspirin-loaded chitosan nanoparticles (ACS) were prepared by Zhang et al., which was contained in an asymmetric collagen-chitosan membrane (CCM). They demonstrated that the ACS-CCM has a higher osteogenic potential due to sustained aspirin release^19^.

Expression of Runx-2 genes which is involved in bone formation and osteoblastic differentiation^68–70^, and OCN gene which is engaged in the mineralization of bone and terminal osteoblastic differentiation^71,72^, were assessed in addition to the expression Runx-2 and OCN proteins to investigate the osteogenic differentiation potential of CCNFs and PACNs membranes.

The Runx-2 gene expression was elevated for both membranes, which may be attributed to the early stages of DPSCs osteogenic differentiation^68^. The elevated OCN expression, which is the most abundant non-collagenous protein in bone ECM, on day 21, may be attributed to the observed profile of Runx-2 gene, because Runx-2 may directly induce the OCN gene transcription^70^. Note that OCN upregulation is critical for the mineralization and terminal osteogenic differentiation^71^. Also, the expression of OCN and RUNX-2 proteins (Fig. 9d, f) may elucidate the enhanced mineralization on CCNFs and PACNs membranes.

**Figure 10:**
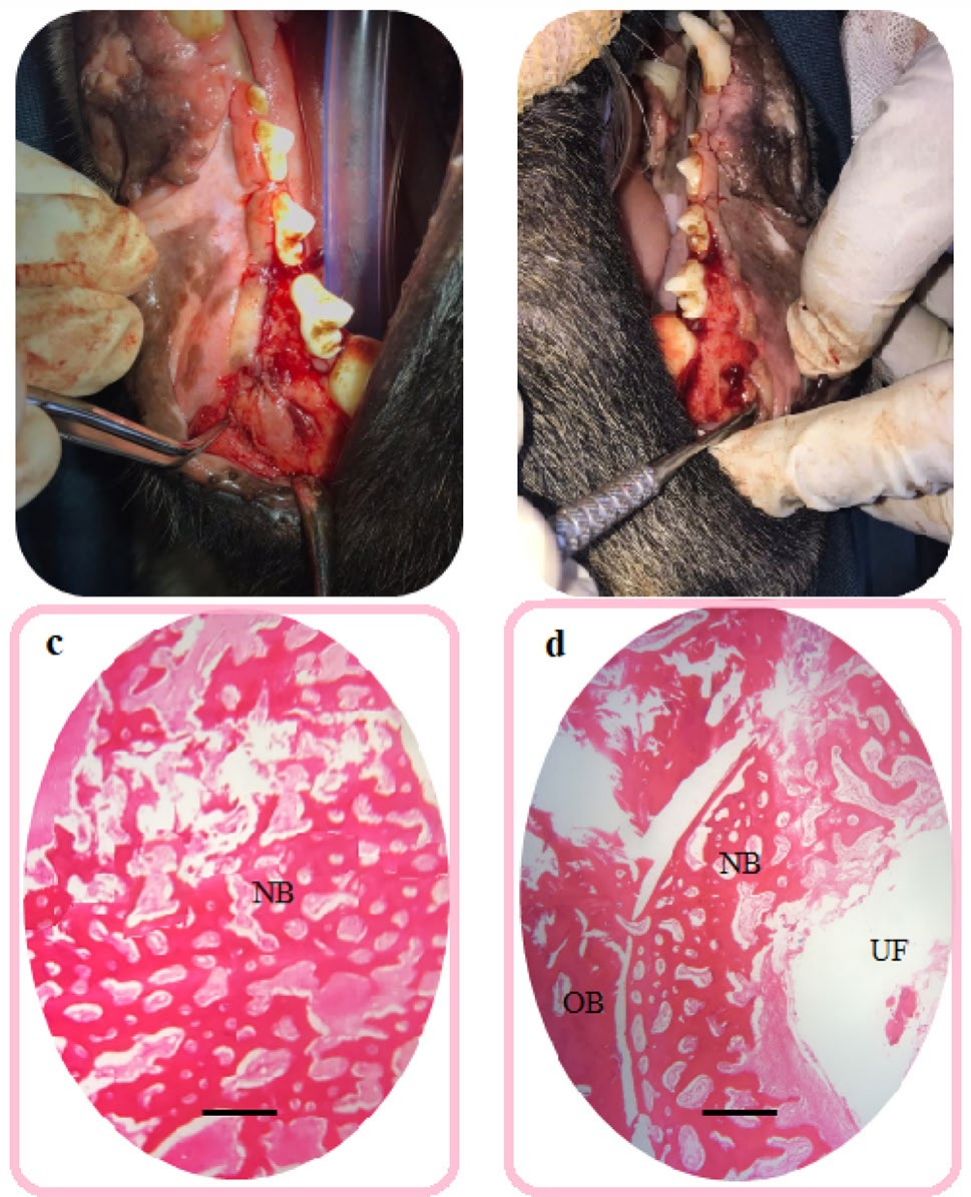
(**a**) the test area (covered with the prepared asymmetric membrane) that was completely filled with new bone, (**b**) the control side (covered with the commercial membrane) that remains empty, (**c**) histological section of bone defects in the test area, (**d**) histological section of bone defects in the control area; *UF* unfilled area, *NB* new bone, *OB* old bone. Original magnification is × 40. Scale bars = 500 µm.

The profile of gene expression of the DPSCs, which were seeded on the membranes control group in the osteogenic media, was consistent with the results of other studies^73,74^. Our findings showed that the new membranes enhanced the osteogenic properties of the collagenic membranes. Increased expression of the osteoblastic genes and associated proteins, besides ALP activity, shows that aspirin and curcumin may enhance the osteogenic differentiation of DPSCs on PACNs and CCNFs electrospun fibers. In vivo findings of the present study also proved that more significant bone formation on the membranes (Fig. 10).

#### In vivo bone regeneration behavior

The results of the animal test showed that the test area (the prepared asymmetric membrane) was completely filled with new bone after just 28 days, while the commercial membrane area remains empty. There was also a soft tissue layer above the new bone area in the test side. No significant inflammatory was detected in the pathology specimens, and the membranes were completely degraded. The results of the histological test (Fig. 10) showed that in the prepared membrane area new bone formation can be seen. In the commercial membrane area, the edges of the defect are still sharp and intact, and in the pathology specimen, the dog’s previous bone can be seen. The newly created bone was observed in the test membrane in HE staining images together the obtained cellular results, suggesting that the PACNFs layer of the membrane acts as osteoinductive material. The CCNFs layer not only can act as a physical barrier for the GBR technique, but also it also has osteocondutive effects (according to cellular results) that improves the PACNFs layer’s action. Besides, the CCNFs layer helps the formation of the soft tissue above the new bone that can be due to wound healing effects of curcumin. Furthermore, the CCNFs side can be used as a local antimicrobial delivery system for avoiding infections in the test surgical area (based on the antimicrobial test’ results.

The main characteristics and the findings for the prepared membrane were summarized in Table 1.

**Table 1:** The main characteristics and the findings for the prepared membrane.

Main finding of tests	Collagen nanofibers (CNFs) including PANPs (PACNFs)	curcumin/collagen nanofibers (CCNFs)
*The asymmetric membrane*		
Physical state	White sheet (thickness of 0.4 mm)	Yellow sheet (thickness of 0.4 mm)
The mean particle size and the surface charge	98.52 nm (PDI of 0.49)	84.06 nm (PDI of 0.39)
	With a negative surface charge of − 32.20 mV	With a negative surface charge of − 37.74 mV
SEM results for morphology	Nanofibers with a network structure and without the presence of beads	Nanofibers with a network structure and without the presence of beads
In vitro cellular examinations	Without any toxic effect on DPSCs	Without any toxic effect on DPSCs
	Increase in proliferation of DPSCs (*p* < 0.05)	Increase in proliferation of DPSCs (*p* < 0.05)
	Increased in ALP activity (*p* < 0.05)	Increased in ALP activity (*p* < 0.05)
	Increase in RNA expression of Runx-2, and OCN as osteogenic genes (*p* < 0.05)	Increase in RNA expression of Runx-2, and OCN as osteogenic genes (*p* < 0.05)
	Increase in protein expression of Runx-2, and OCN as osteogenic genes (*p* < 0.05)	Increase in protein expression of Runx-2, and OCN as osteogenic genes (*p* < 0.05)
Microbial test	Not showed any antimicrobial effects	It showed antibacterial effects against *S. aureus*, *E. faecalis* and *E. coli*
		The membrane was more effective against *S. aureus* followed by *E. faecalis* and *E. coli*
In vivo findings	The asymmetric membrane completely occupied after just 28 days, while the commercial membrane area remains empty	
	No significant inflammatory was detected in the pathology specimens, and the membranes were completely degraded	
	The results of the microscopic examination showed that in the prepared membrane area new bone formation can be seen. The newly created bone was detected in the test membrane in HE staining images, suggesting that the PACNFs layer of the membrane is osteoinductive and that the CCNFs layer not only can act as a physical barrier for the GBR technique, but also it also has osteocondutive effects (according to cellular results) that improves the PACNFs layer’s action	

## Conclusion

The main purpose of this study was to design an asymmetric GBR membrane using electrospinning technique for the sustained release and local delivery of aspirin and curcumin. We hypothesized that the prepared membrane in this work acts as osteoinductive material to promote the new bone formation. Besides, the prepared multipurpose asymmetric membrane not only can keep soft tissue from occupying bone defects in GBR surgeries, but also it benefits from antibacterial activities and bone healing effects of curcumin. In the field of periodontal therapies, bone regeneration remains a more challenging issue. Nano-dentistry is trying its best to apply new signs of progress in tissue engineering and dental practice. With the growth of advanced investigations and deeper understanding of electrospinning sets-ups, it is probable to attain future "smart bone healing devices" proficient in treating all features of bone defects for real clinical uses.

## Supplementary information

Supplementary Figure 1.

## Data Availability

The raw/processed data needed to reproduce these outcomes cannot be shared at this time as the data also are part of an ongoing study.
