# Supplementary material for: Nanofibrous asymmetric collagen/curcumin membrane containing aspirin-loaded PLGA nanoparticles for guided bone regeneration
Source: Sci Rep. 2020 Oct 23;10:18200. doi: 10.1038/s41598-020-75454-2 (PMC7584591; doi:10.1038/s41598-020-75454-2)
Supplement: Supplementary file 1 — Supplementary Figure 1. [file 41598_2020_75454_MOESM1_ESM.pdf]

SUPPLEMENTARY MATERIAL

Nanofibrous asymmetric collagen/curcumin membrane containing aspirin-loaded PLGA nanoparticles for guided bone regeneration

Mohammad Ali Ghavimi [a,#], Amirhossein Bani Shahabadi [a,#] , Seyedhosein Jarolmasjed [b], Mohammad Yousef Memar [c], Solmaz Maleki Dizaj [d,*], Simin Sharifi [d,e,*]

[a] *Department of Oral and Maxillofacial Surgery, Faculty of Dentistry, Tabriz University of Medical Sciences, Tabriz, Iran*

[b] *Department of Clinical Sciences, Faculty of Veterinary Medicine, University of Tabriz, Tabriz, Iran*

[c] *Infectious and Tropical Diseases Research Center, Tabriz University of Medical Sciences, Tabriz, Iran*

[d] *Dental and Periodontal Research Center, Tabriz University of Medical Sciences, Tabriz, Iran*

[e] *Stem Cell Research Center, Tabriz University of Medical Sciences, Tabriz, Iran*

[#] *Co-First/Equal authorship*

*maleki.s.89@gmail.com

P* sharifi.ghazi@gmail.com

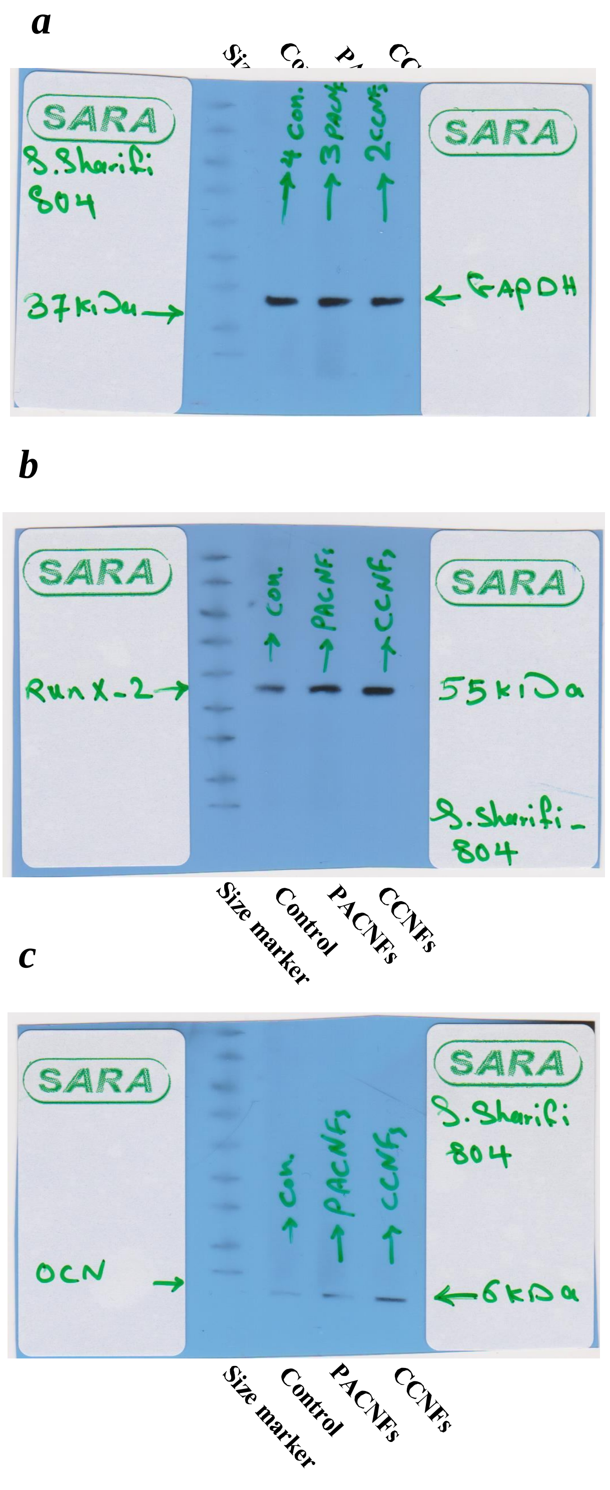

Figure 1. Osteogenic differentiation of DPSCs cultured on PACNs and CCNFs membranes at 21day in the osteogenic medium. The cell lysates were prepared and used for western blot with GAPDH, Runx-2 and OCN antibodies. The figure shows (***a***) GAPDH (37 kDa), (***b***) Runx-2 (55 kDa) (***c***) OCN (6 kDa). The gels were not cropped.
